# Model estimates of hospitalization discharge rates for norovirus gastroenteritis in Europe, 2004–2015

**DOI:** 10.1186/s12879-021-06421-z

**Published:** 2021-08-05

**Authors:** Elsa Negro Calduch, Tom Cattaert, Thomas Verstraeten

**Affiliations:** P95 Pharmacovigilance and Epidemiology Services, Leuven, Belgium

**Keywords:** Norovirus, Acute gastroenteritis, Hospitalization, Europe, Discharge registers, Community-onset

## Abstract

**Background:**

Norovirus is an important cause of acute gastroenteritis globally. However, norovirus is rarely laboratory confirmed or recorded explicitly as a cause of hospitalization. In recent years, there has been an interest in using medical databases and indirect modelling methods to estimate the incidence of norovirus gastroenteritis. The objective of this study was to estimate the incidence of hospitalizations for norovirus gastroenteritis in Europe (2004–2015) using nationwide in-patient discharge records from different European countries.

**Methods:**

National hospital discharge registers in all 28 European Union countries (at that time) and all 4 European Free Trade Association countries were contacted and invited to participate in the study. Discharges with ICD9/ICD10 codes for acute gastroenteritis (AGE) as first-listed (principal) diagnosis were extracted to assess hospitalization rates for AGE and norovirus gastroenteritis (NGE), overall, by age group, country, month, and seasonal year. The number of cause-unspecified episodes was regressed against pathogen-specific AGE episodes: Rotavirus, *Clostridium difficile*, Other Bacterial, Other Viral and Parasitic separately. NGE hospital discharges were estimated for each month by calculating the difference between observed cause-unspecified and model-predicted counts, assuming that any remaining seasonality not otherwise captured in the model was due to norovirus, and adding those to the coded NGE episodes to get the total number of norovirus-associated episodes.

**Results:**

Data were available from 15 countries, representing 68% of the total population in Europe. Only 24.4% of all AGE discharges were coded as cause-specified. We estimated that between 2004 and 2015, the overall rate of NGE hospital discharges in Europe was 3.9 per 10,000 person-years, ranging from 1.2 (Portugal) to 10.7 (Lithuania). Norovirus was predicted to be responsible for 17% of all AGE hospital discharges in Europe in this period. Norovirus affects individuals of all ages, but NGE discharge rates were highest in children < 5 years (24.8 per 10,000 person-years), and adults aged ≥80 years (10.7 per 10,000 person-years).

**Conclusion:**

We estimated that 1 in 400 hospitalizations in Europe can be attributed to Norovirus. In the absence of routine norovirus testing and recording in hospital settings, modelling methods are useful resources to estimate the incidence of norovirus gastroenteritis.

**Supplementary Information:**

The online version contains supplementary material available at 10.1186/s12879-021-06421-z.

## Introduction

Norovirus is an important cause of acute gastroenteritis (AGE) globally across all age groups [1–3]. Populations at increased risk of norovirus gastroenteritis (NGE) are the very young, the immunocompromised, and those living in closed communities such as long-term care facilities [4, 5]. A recent systematic review estimated that norovirus caused 677 million AGE cases worldwide in 2010 [2]. Despite being a major contributor of diarrheal disease worldwide, norovirus is rarely laboratory confirmed or recorded as a cause of hospitalization. As an example, from July 2007 through June 2013 90% of all gastroenteritis hospital discharges in England were of undetermined etiology [6].

To overcome these issues, in recent years there has been an increasing interest and use of medical databases and mathematical models to indirectly estimate the incidence of norovirus-associated disease [7–11]. In-patient datasets are usually recorded as mandatory national health registers in most European countries. These registers contain information on hospital discharges by diagnostic codes commonly using the International Classification of Diseases, Ninth/tenth Revision (ICD-9/10). Upon request, register data holders may release statistics for specific scientific research purposes.

In order to better understand the incidence of NGE hospitalizations in Europe, we conducted a retrospective, observational study of national in-patient discharge registers from 2004 through 2015 of countries in Europe with the aim of estimating the incidence of community-onset AGE and NGE requiring hospitalization in Europe. This information can serve to inform public health decisions related to the potential introduction of norovirus immunization in Europe.

## Methods

National hospital discharge registers in all 28 European Union (EU28) countries and all 4 European Free Trade Association (EFTA) countries were contacted and invited to participate in the study. Procedures for data application were fulfilled as required. Hospital statistics data were included if available upon request without apparent restrictions, with or without charge, and when episode records could be classified by AGE-related ICD-9 or ICD-10 codes (see Supplemental Table 1 for a comparison of the codes in the two systems). As the first-listed ICD9/10 code on discharge records presents the principal diagnosis and hence condition leading to a person being admitted to hospital, analyses were limited to discharge records with AGE as the first listed diagnosis in order to capture the burden of hospitalizations for community-onset AGE. Only finalized discharges of inpatients were included. A hospital discharge was defined as the release of a patient who was hospitalized for a minimum of one night: same-day discharges were excluded. When national registers did or could not release individual data, data aggregated by the following diagnostic categories were requested: norovirus, rotavirus, ‘other viral’, *Clostridium difficile*, ‘other bacterial’, not otherwise specified, and parasitic. The study included all subjects of all ages, and both genders registered in hospital discharge statistics between 2004 through 2015.

Population estimates were obtained from Eurostat for EU and EFTA countries, and from the UK’s Office for National Statistics for England. Denominators were obtained by multiplying the total population estimate by the coverage percentage of the dataset. The representativeness of national hospital statistics was obtained from data providers.

### Ethical considerations

In accordance with the Good Practice Secondary Data Analysis (GPS) guidelines [12], neither ethical committee review nor informed consent was required as it does not apply to secondary data analyses, provided that all the data protection provisions on pseudo-anonymization of personal data are fulfilled and no link to primary data is intended. Secondary data in this study are defined as routine data obtained from population-based hospital registers.

Anonymized data were transferred upon signature of legal agreements to a secure environment within the P95 data management center with access restricted to accredited data analysts. Original copies were destroyed. Occasionally, further data were transferred when discrepancies with the initial request were identified. This study was conducted in accordance with the Declaration of Helsinki [13] and the International Ethical Guidelines on Epidemiological Studies issued by the Council for International Organizations of Medical Sciences (CIOMS) [14].

### Statistical analysis & modelling

Norovirus is not routinely tested and recorded in hospital settings, therefore, we estimated the proportion of cause-unspecified gastroenteritis codes that were attributable to norovirus using negative binomial regression by means of a previously developed statistical model [6–8, 15, 16] that is based on the temporal patterns of occurrence of gastroenteritis.

The expected number of discharges ‘not otherwise specified’ was modelled as:

1 $$ \mathrm{E}\left(\mathrm{N}\_{\mathrm{NOS}}_{\mathrm{x},\mathrm{t}}\right)=\left({\upalpha}_{\mathrm{x}}\times {\mathrm{pt}}_{\mathrm{x},\mathrm{t}}\right)+\left({\upbeta 1}_{\mathrm{x}}\times \mathrm{N}\_{\mathrm{CDIFF}}_{\mathrm{x},\mathrm{t}}\right)+\left({\upbeta 2}_{\mathrm{x}}\times \mathrm{N}\_\mathrm{OTHER}\ {\mathrm{BACTERIAL}}_{\mathrm{x},\mathrm{t}}\right)+\left({\upbeta 3}_{\mathrm{x}}\times \mathrm{N}\_\mathrm{OTHER}\ {\mathrm{VIRAL}}_{\mathrm{x},\mathrm{t}}\right)+\left({\upbeta 4}_{\mathrm{x}}\times \mathrm{N}\_{\mathrm{PARASITIC}}_{\mathrm{x},\mathrm{t}}\right)+\left({\upbeta 5}_{\mathrm{x}}\times \left(\mathrm{N}\_{\mathrm{ROTA}}_{<5,\mathrm{t}}/\mathrm{pt}.{}_{<5,\mathrm{t}}\right)\times {\mathrm{pt}}_{\mathrm{x},\mathrm{t}}\right)+\left({\upgamma}_{\mathrm{x}}\times \mathrm{t}\times {\mathrm{pt}}_{\mathrm{x},\mathrm{t}}\right) $$

Variables with negative coefficients were removed regardless of statistical significance, because they are impossible in the additive interpretation of the model, with the temporal pattern of the cause-unspecified AGE being expressed as superposition of the temporal patterns of AGE due to the various specific causes.

N represents counts of AGE episodes (E) due to causes ‘not otherwise specified’ (NOS), *Clostridium difficile* (CDIFF), other bacterial infections (OTHER BACTERIAL), other viral infections (OTHER VIRAL), parasitic infections (PARASITIC) or rotavirus (ROTA), for age group x, and month t. Not otherwise specified (NOS) gastroenteritis was defined on the basis of the following ICD-10-CM codes recorded in the first diagnostic position: other specified and unspecified bacterial intestinal infections (A04.8, A04.9), other specified and unspecified foodborne intoxications (A05.8, A05.9), other specified and unspecified intestinal infection (A08.3, A08.4, A08.5), infectious gastroenteritis and colitis of unspecified origin (A09.0-A09.9), presumed non-infective gastroenteritis and colitis (K52.8, K52.9) and diarrhea (R19.7). *α* represented the background discharge rate not explained by infections due to one of the specified pathogen categories; β represented specific causes; and γ represented a secular linear time trend adding to the background rate. Person-time (in days) for age group x during month t was expressed as pt_x,t_.

Rotavirus infection counts in the 0–4-year age group were used as the predictor for all age groups because laboratory confirmation of rotavirus infection is generally limited to this age group and is rare outside pediatric populations. Backward stepwise regression was employed; variables that were not significant at the 5% level or those with negative coefficients were removed for each age group x separately.

Since norovirus disease is rarely recorded, coded norovirus discharges were not included in the equation. Instead, and as previously applied in other studies [6–8, 15–17], NGE discharges were estimated for each month by calculating the difference between observed cause-unspecified and model-predicted counts, i.e. the model residuals r_x,t_ = N_NOS_x,t_ – E(N_NOS_x,t_), assuming that any remaining seasonality not otherwise captured in the model was due to norovirus. The (negative) quantity pt_x,t_ × min (r_x,t_/pt_x,t_) was subtracted from these residuals and added to the background, assuming that there is a month in which there are no NGE discharges.

The minimum represented the seasonal (July to June) minimum norovirus rate based on the raw residuals. Estimated NGE discharges modelled from nonspecific episodes were then added to the coded NGE discharges to get the total number of NGE discharges. Totals over the various countries included in the study were obtained afterwards.

Category-specific hospital discharge rates per 10,000 person-years and exact Poisson confidence interval were calculated using annual population estimates obtained from Eurostat from 2004 through 2015 as denominators [18]. Variation across countries in the population covered by the respective discharge databases was taken into account and denominators were adjusted accordingly. Every estimate reported is based only on data available, e.g. some countries did not contribute for the entire study period, 2004–15. Discharge rates were calculated by etiology, age group, month, seasonal year and country.

The denominator was adjusted to the total coverage taking into account the share of discharges covered by each register (i.e. publicly funded hospitals) out of the total discharges (i.e. private & publicly funded). This share was multiplied by the total mid-year population in each country. We made two assumptions, namely, the proportion of discharges in public and private institutes of the total health institutions was the same for related AGE diagnosis (in other words there is no preferential hospitalization of AGE cases in the public or private sector) and secondly, the proportion of the population covered corresponds to percentage of total discharges in the health institutions covered by the register (private/public).

The rate of predicted norovirus hospital discharges per 100,000 inhabitants was calculated by multiplying predicted age-specific rates by population of EU28 and EFTA countries for 2004–2015. We also assumed that data obtained was representative of the 32 European countries as the countries for which we estimated NGE represent 68% of the total population and are geographically widely spread.

SAS Proprietary Software version 9.4, Copyright by SAS Institute Inc., was used for data management and descriptive statistics and R v.3.3.1 was used for visualization and modelling.

## Results

### Description of data sources

National hospital discharge statistics were obtained from 16 EU & EFTA countries: Austria, Cyprus, Denmark, England (not UK), Finland, Germany, Hungary, Italy, Lithuania, Malta, Norway, Poland, Portugal, Romania, Spain, and Sweden (Supplemental Table 2). Data from Norway were excluded from this analysis because first-listed and secondary diagnosis were aggregated. Finland provided data aggregated for the age groups < 18 years, 18–59 years, and ≥ 60 years. For Spain, Austria, and Cyprus data were available from 2004 through to 2014, for Italy from 2004 through to 2013, for Romania from 2006 through to 2015, and for Malta from 2010 through to 2015. No data were obtained from other EU & EFTA countries for reasons explained in Supplemental Table 3. Based on Eurostat reports, data for the 15 EU & EFTA countries for which AGE discharge data were included in this analysis, represented approximately 68% of the total population as well as approximately 68% of all hospital discharges in the 32 EU & EFTA countries in this period.

### Observed all-cause gastroenteritis discharge rates

From 2004 through 2015, the annual mean number of discharges for all-cause AGE across the 15 included European countries was 771,834 per year. This corresponds to an annual mean discharge rate of 23.3 [95% confidence interval [CI], 23.3–23.3] per 10,000 person-years. There was a wide variation between countries: mean annual discharge rates for all-cause AGE were the highest at 55.0 per 10,000 person-years in Lithuania, followed by Austria (42.7) and Germany (39.4), and were the lowest in Hungary (7.2), Italy (8.6), Portugal (10.8) and Spain (10.9) (Supplemental Table 4).

**Fig. 1:**
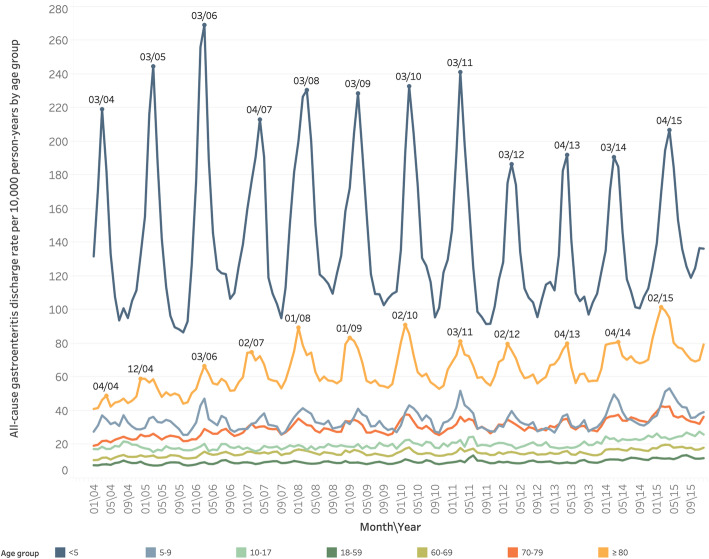
Seasonal and Long-Term Trends of All-cause Gastroenteritis, by Age, in Discharges per 10,000 person-years, in Europe (15 countries), 2004–2015

Figure 1 shows observed age-specific all-cause AGE discharge rates per 10,000 person-years for the 15 included European countries. The highest all-cause AGE discharge rates were observed in children aged < 5 years, with an annual mean discharge rate of 142.6 [95% CI, 142.5–142.8] per 10,000 person-years, followed by elderly adults ≥80 years with 65.6 [95% CI, 65.5–65.7] discharges per 10,000 person-years.

All-cause AGE discharge rates per 10,000 person-years increased by 34.0% from 20.9 [95% CI, 20.9–21.0] in season 2004/05 to 28.0 [95% CI, 27.9–28.0] in season 2014/15 in all ages. The highest increase was observed in elderly adults ≥80 years, with AGE rates increasing by 63.4% from 51.6 per 10,000 person-years in season 2004/05 to 84.3 per 10,000 person-years in season 2014/15.

A clear seasonal pattern in all-cause AGE discharge rates was observed, with annual peaks in March–April. This pattern was most marked among children aged < 5 years, and to a lesser degree in elderly adults aged ≥80 years and children aged 5-to-9-years (Fig. 1).

Most AGE discharges were of undetermined etiology (75.5%; Table 1). Cyprus (97.1%) and Malta (96.6%) had the highest proportion of cause-unspecified AGE hospital discharges, and Lithuania (61.8%) and Sweden (69.6%) the lowest. Cause-unspecified AGE hospital discharges increased by 21.1% from 16.6 [95% CI, 16.6–16.6] per 10,000 person-years in season 2004/05 to 20.1 [95% CI, 20.1–20.2] per 10,000 person-years in season 2014/15.

**Table 1:** Observed Annual Mean Number and Percentage of Total All-Cause Gastroenteritis of In-Patient Discharges by Pathogen Category in primary diagnostic position (15 European countries), 2004–2015

	Annual mean number and percentage of gastroenteritis discharges by pathogen category							
Country	Norovirus^a^	Rotavirus^b^	*Clostridium difficile*^c^	Parasitic^d^	Other bacterial^e^	Other viral^f^	Cause unspecified/NOS^g^	All-cause gastroenteritis
Austria	572 (1.6%)	1651 (4.6%)	1015 (2.9%)	35 (0.1%)	1560 (4.4%)	261 (0.7%)	30,464 (85.7%)	35,557 (100%)
Cyprus	0 (0.0%)	0 (0.0%)	0 (0.0%)	2 (0.2%)	27 (2.7%)	0 (0.0%)	953 (97.1%)	982 (100%)
Denmark	116 (1.0%)	186 (1.6%)	773 (6.5%)	30 (0.2%)	388 (3.2%)	18 (0.1%)	10,460 (87.4%)	11,971 (100%)
England	562 (0.7%)	1582 (1.9%)	6005 (7.3%)	191 (0.2%)	3463 (4.2%)	83 (0.1%)	70,731 (85.6%)	82,618 (100%)
Finland	55 (0.4%)	505 (3.9%)	1567 (12.1%)	45 (0.3%)	386 (3.0%)	91 (0.7%)	10,346 (79.6%)	12,995 (100%)
Germany	22,934 (7.1%)	25,009 (7.8%)	23,096 (7.2%)	551 (0.2%)	20,921 (6.5%)	3243 (1.0%)	225,809 (70.2%)	321,564 (100%)
Hungary	2 (0.0%)	328 (4.7%)	326 (4.6%)	10 (0.1%)	575 (8.2%)	28 (0.4%)	5768 (82.0%)	7035 (100%)
Italy	81 (0.2%)	4990 (10.0%)	1557 (3.1%)	355 (0.7%)	4267 (8.5%)	622 (1.2%)	38,100 (76.2%)	49,972 (100%)
Lithuania	460 (2.7%)	3387 (19.5%)	30 (0.2%)	13 (0.1%)	2607 (15.0%)	132 (0.8%)	10,717 (61.8%)	17,347 (100%)
Malta	2 (0.1%)	4 (0.3%)	6 (0.5%)	0 (0.0%)	31 (2.5%)	0 (0.0%)	1200 (96.6%)	1243 (100%)
Poland	159 (0.2%)	16,868 (16.1%)	1939 (1.8%)	980 (0.9%)	5786 (5.5%)	1412 (1.3%)	77,862 (74.1%)	105,006 (100%)
Portugal	3 (0.0%)	581 (6.7%)	398 (4.6%)	13 (0.1%)	481 (5.6%)	50 (0.6%)	7088 (82.3%)	8614 (100%)
Romania	15 (0.0%)	2049 (3.5%)	1061 (1.8%)	1475 (2.5%)	2613 (4.5%)	131 (0.2%)	51,311 (87.5%)	58,656 (100%)
Spain	13 (0.0%)	3814 (8.7%)	1120 (2.6%)	162 (0.4%)	4941 (11.3%)	329 (0.7%)	33,476 (76.3%)	43,856 (100%)
Sweden	832 (5.8%)	523 (3.6%)	1783 (12.4%)	46 (0.3%)	1146 (7.9%)	49 (0.3%)	10,040 (69.6%)	14,419 (100%)
All	25,807 (3.5%)	61,478 (8.0%)	40,676 (5.4%)	3909 (0.5%)	49,191 (6.4%)	6450 (0.8%)	584,324 (75.5%)	771,834 (100%)

NOTE. Annual mean number of discharges and percentage of total all-cause gastroenteritis per pathogen category

*NOS* not otherwise specified

^a^ICD-10 codes A08.1; A08.11; A08.19; A08.31

^b^ICD-10 code A08.0

^c^ICD-10 code A04.7

^d^ICD-10 codes A06; A06.0; A06.1; A06.2; A06.3; A06.8; A06.9; A07

^e^ICD-10 codes A00; A01; A02; A03; A04.0; A04.1; A04.2; A04.3; A04.4; A04.5; A04.6; A05.0; A05.1; A05.2; A05.3; A05.4; A05.5

^f^ICD-10 codes A08.2; A08.32

^g^ICD-10 codes A04; A04.8; A04.9; A05; A05.8; A05.9; A08; A08.3; A08.39; A08.4; A08.5; A08.8; A09; K52.8; K52.9; R19.7

### Predicted norovirus discharge rates

As the number of reported pathogen-specific AGE discharges were insufficient for Malta and Cyprus we could not achieve model convergence in these two countries. The goodness of fit of the model for the remaining countries is shown in Supplemental Table 5.

**Fig. 2:**
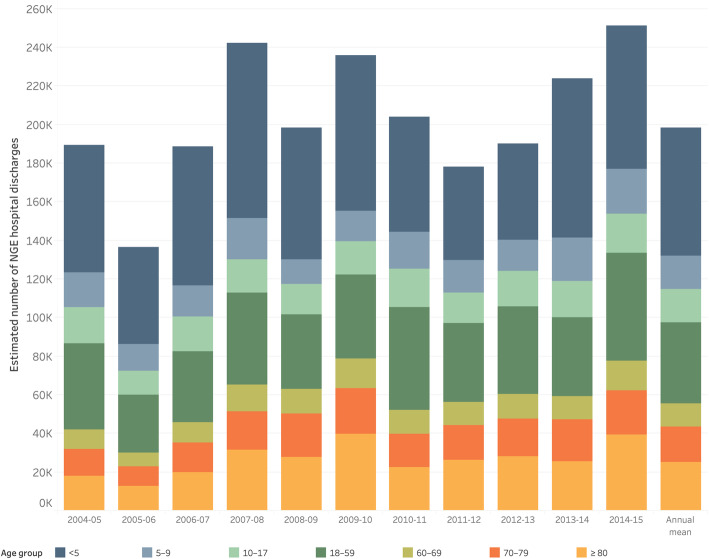
Estimated Number of Norovirus-associated Hospital Discharges by Age Group and Season, 2004–2015, in EFTA and EU countries (*n* = 32)

We estimated that on average there were 199,087 hospital discharges attributable to norovirus per year in Europe (*n* = 32 countries), of which 130,608 were in the 13 countries included in this analysis. It was estimated that 33.4% of NGE hospital discharges occurred in children aged < 5 years (mean of 66,459 discharges per year), and 27.8% in adults aged > 60 years (mean of 55,321 discharges per year). The highest number of NGE discharges was predicted in seasons 2014/15 (247,640), 2007/08 (245,564), 2009/10 (235,549), and 2013/14 (219,157), which corresponds with an increase above the annual mean of 24.4, 23.4, 18.3, and 10.0% respectively (Fig. 2).

Norovirus was estimated to have caused 16.7% of AGE discharges in all ages, 17.5% in children aged < 5 years, and 15.8% in adults aged > 60 years. Comparing the last 5 seasons to the first 6 seasons, the relative contribution of norovirus to AGE declined modestly from 17.4 to 16.3%.

**Fig. 3:**
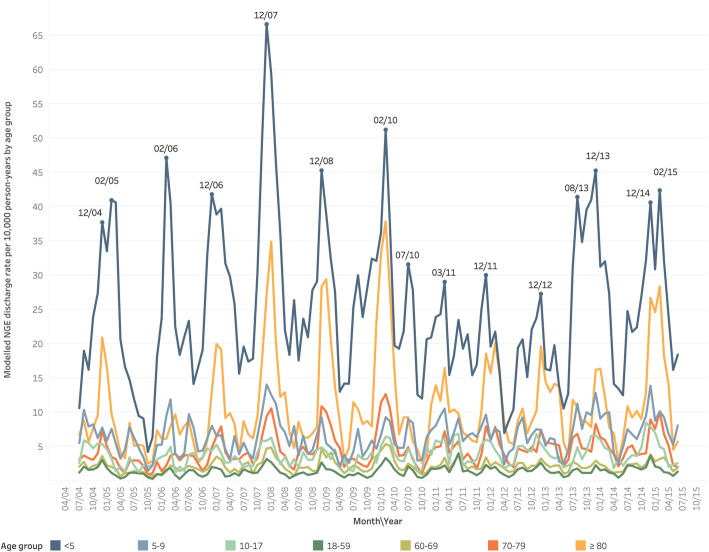
Seasonal and Long-Term Trends of NGE, by Age, in Discharges per 10,000 person-years in Europe (13 countries), 2004–2015

The annual rate of NGE-related hospital discharges in Europe was estimated at 3.9 [95% CI, 3.8–3.9] per 10,000 person-years in all ages, with the highest rates in children aged < 5 years (24.8, 95% CI, 24.1–25.4) and elderly adults aged ≥80 years (10.7, 95% CI, 10.4–11.1) (Table 2). Estimated NGE discharge rates varied widely between participating countries: rates were the highest in Lithuania (10.7 per 10,000 person-years), followed by Finland (8.5), Austria (7.9), Germany (6.9), and Romania (6.7), and the lowest in Italy (1.2), Spain (1.4), Hungary (1.7), England (1.8) and Portugal (2.5) (Table 3). A clear seasonal pattern was observed with NGE discharge rates peaking during the winter months. Seasonality of NGE was evident among children aged < 5 years and to a lesser extent in elderly adults aged ≥80 years and children aged 5–9-years (Fig. 3). In July 2010 and August 2013, peaks in NGE discharges outside the expected winter period were observed.

**Table 2:** Estimated Community-Acquired Norovirus Hospital Discharge Rate and 95% CI by Age Group and Year in Europe (13 countries), 2004–2015

	Age group, NGE discharge rate per 10,000 person-years (95% CI)							
Year	< 5 years	5–9 years	10–17 years	18–59 years	60–69 years	70–79 years	≥ 80 years	All ages
2004–05	25.1 (23.6–26.8)	6.8 (6.0–7.5)	3.9 (3.4–4.3)	1.5 (1.4–1.6)	2.0 (1.7–2.3)	3.6 (3.0–4.1)	9.1 (8.1–10.2)	3.8 (3.7–4.0)
2005–06	19.3 (17.9–20.8)	5.2 (4.5–5.8)	2.6 (2.2–3.0)	1.0 (0.9–1.1)	1.4 (1.1–1.6)	2.6 (2.2–3.1)	6.2 (5.2–7.4)	2.9 (2.8–3.0)
2006–07	27.4 (24.8–30.5)	6.1 (5.3–6.9)	3.8 (3.3–4.3)	1.2 (1.1–1.4)	2.1 (1.8–2.4)	3.9 (3.4–4.4)	9.2 (8.2–10.3)	3.7 (3.6–3.9)
2007–08	34.2 (31.9–36.8)	8.0 (7.2–9.0)	3.7 (3.3–4.2)	1.6 (1.5–1.8)	2.6 (2.3–2.9)	5.0 (4.6–5.5)	14.1 (12.9–15.7)	4.8 (4.6–5.0)
2008–09	25.4 (23.4–27.7)	4.9 (4.2–5.6)	3.4 (3.0–3.9)	1.3 (1.2–1.4)	2.4 (2.1–2.7)	5.6 (5.0–6.3)	12.1 (11.0–13.4)	3.9 (3.7–4.1)
2009–10	29.7 (27.6–32.0)	5.9 (5.2–6.7)	3.9 (3.4–4.4)	1.5 (1.3–1.6)	2.8 (2.5–3.1)	5.8 (5.3–6.4)	16.7 (15.1–18.4)	4.6 (4.4–4.7)
2010–11	21.7 (19.9–23.9)	7.3 (6.6–8.0)	4.5 (4.1–5.0)	1.8 (1.7–1.9)	2.2 (2.0–2.5)	4.2 (3.8–4.7)	9.2 (8.3–10.3)	3.9 (3.7–4.1)
2011–12	17.7 (15.7–19.7)	6.3 (5.6–7.1)	3.6 (3.2–4.1)	1.4 (1.3–1.5)	2.1 (1.9–2.4)	4.3 (3.9–4.8)	10.4 (9.4–11.6)	3.4 (3.3–3.6)
2012–13	18.2 (16.6–20.0)	6.0 (5.3–6.9)	4.2 (3.7–4.8)	1.5 (1.4–1.7)	2.2 (1.9–2.5)	4.8 (4.2–5.3)	10.8 (9.8–12.0)	3.7 (3.5–3.8)
2013–14	30.4 (27.8–33.3)	8.3 (7.5–9.3)	4.3 (3.8–4.9)	1.4 (1.2–1.6)	2.0 (1.8–2.4)	5.2 (4.5–5.8)	9.7 (8.6–11.1)	4.2 (4.0–4.4)
2014–15	27.6 (25.1–30.7)	8.4 (7.3–9.6)	4.7 (4.1–5.3)	1.9 (1.7–2.1)	2.6 (2.2–3.0)	5.4 (4.8–6.2)	14.5 (12.8–16.4)	4.8 (4.5–5.0)
Annual mean	24.8 (24.1–25.4)	6.5 (6.3–6.8)	3.8 (3.6–3.9)	1.4 (1.4–1.5)	2.2 (2.1–2.3)	4.5 (4.3–4.6)	10.7 (10.4–11.1)	3.9 (3.8–3.9)

*CI* confidence interval, *NGE* norovirus-associated gastroenteritis

**Table 3:** Estimated Annual Mean of Community-Acquired Norovirus-Associated Hospital Discharge Rate and 95% CI by Age Group and Country, 2004–2015

	Age group, NGE discharge rate per 10,000 person-years (95% CI)							
Year	< 5 years	5–9 years	10–17 years	18–59 years	60–69 years	70–79 years	≥ 80 years	All ages
Austria	45.7 (41.6–50.3)	13.8 (12.3–15.4)	7.4 (6.5–8.5)	3.4 (3.1–3.7)	4.6 (3.9–5.3)	10.4 (9.1–11.8)	23.5 (20.8–26.3)	7.9 (7.5–8.2)
Denmark	37.4 (33.6–41.7)	5.1 (4.3–5.9)	2.7 (2.3–3.2)	2.0 (1.8–2.3)	3.5 (2.9–4.1)	7.5 (6.4–8.7)	16.0 (14–18.2)	5.5 (5.1–5.8)
England	8.6 (7.6–9.7)	1.5 (1.3–1.7)	0.9 (0.8–1.1)	0.6 (0.6–0.7)	1.4 (1.2–1.6)	3.0 (2.6–3.4)	8.1 (7.3–8.9)	1.8 (1.7–1.9)
Finland	n/a	n/a	n/a	1.9 (1.7–2.1)	n/a	n/a	n/a	8.5 (7.9–9.2)
Germany	41.1 (39.8–42.4)	9.5 (9.0–10.0)	6.8 (6.5–7.2)	3.3 (3.1–3.4)	3.7 (3.4–3.9)	8.2 (7.8–8.6)	22.4 (21.4–23.5)	6.9 (6.8–7.0)
Hungary	15.4 (13.6–17.4)	5.0 (4.3–5.7)	2.3 (1.9–2.6)	0.4 (0.4–0.5)	0.8 (0.7–0.9)	1.5 (1.3–1.8)	2.6 (2.2–3.1)	1.7 (1.6–1.9)
Italy	7.5 (6.8–8.3)	2.4 (2.1–2.8)	1.1 (1.0–1.3)	0.4 (0.4–0.5)	0.8 (0.6–0.9)	1.2 (1.0–1.4)	2.8 (2.4–3.2)	1.2 (1.1–1.2)
Lithuania	87.6 (80.5–94.3)	32.6 (29.4–36)	16.5 (15–18.1)	2.9 (2.6–3.3)	4.4 (3.7–5.2)	8.0 (6.9–9.1)	11.8 (10–13.6)	10.7 (10.2–11.1)
Poland	35.6 (33.6–37.5)	12.4 (11.4–13.5)	5.7 (5.2–6.3)	0.7 (0.6–0.8)	1.5 (1.3–1.8)	2.6 (2.2–3)	4.8 (4.2–5.5)	3.8 (3.7–4)
Portugal	21.0 (18.6–23.6)	6.0 (5.1–7)	2.6 (2.2–3.1)	0.5 (0.4–0.6)	1.6 (1.3–1.9)	2.7 (2.3–3.2)	6.1 (5.1–7.1)	2.5 (2.4–2.7)
Romania	58.4 (51.7–66)	15.9 (13.7–18.3)	7.2 (6.2–8.4)	2.0 (1.8–2.3)	3.7 (3.2–4.2)	5.5 (4.7–6.4)	6.3 (5.5–7.2)	6.7 (6.3–7.1)
Spain	10.7 (9.7–11.8)	1.9 (1.6–2.2)	0.8 (0.7–1)	0.3 (0.3–0.4)	1.3 (1.1–1.5)	2.5 (2.1–2.8)	4.5 (3.8–5.1)	1.4 (1.4–1.5)
Sweden	28.5 (24.1–33.4)	4.1 (3.4–4.9)	1.3 (1.1–1.5)	1.3 (1.1–1.4)	2.8 (2.5–3.1)	5.2 (4.6–5.9)	20.0 (18.4–21.8)	4.4 (4.1–4.8)
All	24.8 (24.1–25.4)	6.5 (6.3–6.8)	3.8 (3.6–3.9)	1.4 (1.4–1.5)	2.2 (2.1–2.3)	4.5 (4.3–4.6)	10.7 (10.4–11.1)	3.9 (3.8–3.9)

Estimated NGE discharge rates per 10,000 person-years (95% CI) in individuals under 18 years old were 13.7 (11.8–15.7) and 19.3 (17.9–21.1) in individuals 60 years and older

*CI* confidence interval, *NGE* norovirus-associated gastroenteritis, *n/a* not available

## Discussion

Using national in-patient registers from 15 European countries, we estimated the incidence of all-cause gastroenteritis and norovirus-associated gastroenteritis leading to hospitalization in Europe from 2004 to 2015. The lack of etiologic data for the cause of gastroenteritis in more than three-quarters of in-patients was addressed by using an indirect method as previously applied [6–11, 17]. We estimated that nearly 200,000 hospital discharges are attributable to norovirus every year across the 32 EU and EFTA countries. This corresponds to an annual NGE hospital discharge rate of 3.9 per 10,000 population.

Few studies have been carried out to assess the incidence of hospitalizations for NGE in Europe. Kowalzik et al. [19] calculated the NGE hospitalization rate in Germany based on aggregate data for hospital discharges with NGE as first-listed diagnosis from the German Federal Statistics Office for 2007–12. Our observed NGE coded hospital discharge rates for the same period match perfectly, as was to be expected when utilizing the same data source. However, when applying the modelling approach, we estimated that the incidence of NGE hospitalizations in Germany in this period was 2.4-fold higher (56,000 predicted compared with 22,000 observed). This suggests a significant underreporting of NGE hospitalizations, even in Germany where testing for norovirus and diagnosis of norovirus as a cause of AGE is relatively high (7.1% of all AGE hospitalizations) compared with other European countries.

The comparability with previous studies that used modelling approaches to estimate the incidence of NGE hospitalizations is limited due to the differing methods used. In studies conducted in England [6] and the United States (US) [8] all discharge records coded with a gastroenteritis diagnosis were considered, irrespective of whether this was the principal or secondary diagnosis. As our aim was to estimate community-onset norovirus hospitalizations, we only included AGE diagnostic codes in the first-listed (principal) position. In an earlier study we performed on the incidence of NGE in England, using a similar modeling method and using data from the Clinical Practice Research Datalink (CPRD) linked to the Hospital Episode Statistics (HES) database, we found a rate of 7.1 NGE hospital discharges per 10,000 person-years [6], compared with 1.8 per 10,000 using only the HES database in the current study. The main differences between the previous CPRD study and current study are that in the first we included all discharges with an AGE code irrespective of whether this was the principal or a secondary diagnoses, thus counting both community-onset and hospital-acquired norovirus infections and including day-cases, while in the current study we only included discharges with AGE as principal diagnosis and defined hospital discharges as the release of a patient who stayed hospitalized for a minimum of one night, which represents 47.3% of all hospitalizations for all causes in the UK [20].

Our study estimated that norovirus was responsible for 17% of all AGE hospital discharges in Europe from 2004 through 2015. These results are in line with the findings of a large systematic literature review that estimated that norovirus was associated with 18% [95% CI, 17–20] of all diarrheal disease worldwide in any setting, and 17% [95% CI, 15–19] specifically in in-patient settings [21].

We found a wide variation in NGE discharge rates between European countries included in this analysis, with a 9-fold difference between the lowest rate in Italy and the highest rate in Lithuania. These differences can be explained to a certain extent by differences in overall hospitalization rates between countries: namely, when comparing estimated NGE hospitalization rates with overall hospitalization rates as reported to Eurostat [20], we found that Lithuania and Romania had the highest estimated NGE discharge rates in children aged < 5 years (87.6 and 58.4 per 10,000 person-years, respectively) and also had the highest overall hospital discharge rates in that age group (4454 and 3817 discharges per 10,000 children aged < 5 years, respectively). Similarly, Austria and Germany had the highest estimated NGE discharge rates in elderly adults aged 80 ≥ years (23.5 and 22.4 per 10,000, respectively) and also had the highest overall hospital discharge rates in this age group (8731 and 6685 discharges per 10,000 elderly). Another source of variability between the estimated NGE rates may be related to the completeness and accuracy of the hospital discharge registers upon which we relied, as well as the robustness of the norovirus coding used in the models.

We estimated a higher number of norovirus discharges during the 2014/15 season followed by seasons 2007/08, 2009/10 and 2013/14. The 2007/08 and 2009/10 peaks coincide with the emergence of new pandemic GII.4 strains (Den Haag 2006B [22, 23] and New Orleans 2009 [24, 25], while the later peaks in 2013–2015 are likely related to the Sydney 2012 strain. The Sydney 2012 strain was originally identified in Australia and was first noted in Europe in the winter of 2012 at relatively low levels [26, 27]. Our data suggest that this strain may have had a delayed impact in Europe.

Our models further suggest that, as expected, hospital discharges for AGE attributable to norovirus peaked during the winter seasons [4, 28, 29]; however, for some of the more recent years a weaker seasonality was observed, with a few distinct peaks of NGE discharges during summer months. The magnitude of these summer peaks varied. Specifically, our model estimated a sharp increase in NGE discharge rates in the summers of 2010 and 2013. As previously suggested by others, this rather atypical increase in norovirus activity during the summer may be associated with the emergence of new pandemic strains, [26, 28, 30–32]. Escaping population immunity may result in atypical seasonality, but this would depend on when in the norovirus season the emerging strain first appeared [28].

Some limitations need to be taken into consideration when interpreting our results. Firstly, the statistical methods applied assumed that any residual seasonality not otherwise captured in the model was due to norovirus, which may lead to an overestimation of norovirus-associated discharge rates. Secondly, to estimate the rate of community-onset norovirus infections requiring hospitalization, we limited our analyses to discharges with AGE codes in the first position. Although the first-listed diagnostic code is considered the principal diagnosis, or medical condition for which a person is admitted to hospital, we cannot exclude that discharges with AGE as secondary diagnosis may have included cases where AGE was in fact the principal reason for hospitalization, and hence we may have underestimated the true incidence of hospitalizations for community-onset norovirus infections. Lastly, we assumed that the countries for which we obtained data are representative of the 32 countries in Europe. We believe this is a fair assumption as the countries for which we estimated NGE represent 68% of the total population and are geographically widely spread.

## Conclusions

By using routinely collected hospital discharge records from a range of European countries, we estimated an overall hospital discharge rate for NGE of 3.9 per 10,000 person-years, or 1 in 400 hospital discharges, in Europe. Norovirus affects all ages, but estimated NGE discharge rates were the highest for children aged < 5 and older adults aged ≥80 years. Estimated NGE discharge rates varied between included European countries, which may be partly explained by countries having different data collection, coding and hospitalization practices.

Our study demonstrates that the burden of NGE on hospitals may be underestimated. Norovirus is a common cause of severe gastroenteritis requiring hospitalization. Increased diagnostic testing for norovirus in hospital settings is needed to better assess the incidence of the disease and to monitor the changing epidemiology of the virus. This information can serve to inform public health decisions related to the health and economic benefit of the potential introduction of norovirus immunization in Europe and to measure the impact of its introduction.

## Supplementary Information

**Additional file 1.** Table S1. Clinical coding grouping. Table S2. Registers that provided national hospital discharge statistics to the present study. Table S3. Non-included countries in Europe: data provider contacted and reason for exclusion. Table S4. Mean annual number and mean annual rate of hospital discharges for all-cause acute gastroenteritis as first-listed diagnosis in participating European countries between 2004 and 2015. Table S5. Goodness of fit. Table S6. List of Data Providers / Requirements.

## Data Availability

P95 adheres to the BMC Infectious Diseases policies on sharing data. However, P95 is legally bound to non-disclosure agreements with data providers. Data can be requested form the data providers, see Table S6 for contact details.

## Abbreviations

AGE: Acute gastroenteritis
CI: Confidence interval
CIOMS: Council for International Organizations of Medical Sciences
EFTA: European Free Trade Association
ICD-9/10: International Classification of Diseases, Ninth/tenth Revision
NGE: Norovirus gastroenteritis
NOS: Not otherwise specified
