# Correction: Model estimates of hospitalization discharge rates for norovirus gastroenteritis in Europe, 2004–2015

**DOI:** 10.1186/s12879-026-13006-1

**Published:** 2026-03-17

**Authors:** Elsa Negro-Calduch, Tom Cattaert, Thomas Verstraeten

**Affiliations:** P95 Pharmacovigilance and Epidemiology Services, Leuven, Belgium

**Correction: BMC Infectious Diseases (2021) 21:757**

10.1186/s12879-021-06421-z

In the original version of this article, the given and family names of Elsa Negro-Calduch were incorrectly structured. The name was displayed correctly in all versions at the time of publication.

The original article has been corrected.

