# Supplementary material for: Model estimates of hospitalization discharge rates for norovirus gastroenteritis in Europe, 2004–2015
Source: BMC Infect Dis. 2021 Aug 5;21:757. doi: 10.1186/s12879-021-06421-z (PMC8340375; doi:10.1186/s12879-021-06421-z)
Supplement: Supplementary file 1 — Additional file 1. Table S1. Clinical coding grouping. Table S2. Registers that provided national hospital discharge statistics to the present study. Table S3. Non-included countries in Europe: data provider contacted and reason for exclusion. Table S4. Mean annual number and mean annual rate of hospital discharges for all-cause acute gastroenteritis as first-listed diagnosis in participating European countries between 2004 and 2015. Table S5. Goodness of fit. Table S6. List of Data Providers / Requirements. [file 12879_2021_6421_MOESM1_ESM.docx]

Model estimates of hospitalization discharge rates for norovirus gastroenteritis in Europe, 2004-2015

Supplemental tables

Table of Contents

[Supplementary Table 1: Clinical Coding Grouping 2](#_Toc62801681)

[Supplementary Table 2: Registers that provided national hospital discharge statistics to the present study 4](#_Toc62801682)

[Supplementary Table 3: Non-included countries in Europe: data provider contacted and reason for exclusion 6](#_Toc62801683)

[Supplementary Table 4: Mean annual number and mean annual rate of hospital discharges for all-cause acute gastroenteritis (AGE) as first-listed diagnosis in participating European countries between 2004 and 2015 7](#_Toc62801684)

[Supplementary Table 5: Goodness of fit 8](#_Toc62801685)

[Supplementary Table 6: List of Data Providers / Requirements 13](#_Toc62801686)

# Supplementary Table 1: Clinical Coding Grouping

|  | | | |
| --- | --- | --- | --- |
| ICD-10 Code | ICD-9 Code | ICD-10 code description | Diagnosis grouping |
| A00 | 001 | Cholera (A00.0; A00.1; A00.9) | OTHER BACTERIAL |
| A01 | 002 | Typhoid and paratyphoid fevers (A01.0; A01.1; A01.2; A01.3; A01.4) | OTHER BACTERIAL |
| A02 | 003 | Other salmonella infections (A02.0; A02.1; A02.2; A02.8; A02.9) | OTHER BACTERIAL |
| A03 | 004 | Shigellosis (A03.0; A03.1; A03.2; A03.3; A03.8; A03.9) | OTHER BACTERIAL |
| A04 |  | Other bacterial intestinal infections | NOS |
| A04.0 | 008.01 | Enteropathogenic Escherichia coli infection | OTHER BACTERIAL |
| A04.1 | 008.02 | Enterotoxigenic Escherichia coli infection | OTHER BACTERIAL |
| A04.2 | 008.03 | Enteroinvasive Escherichia coli infection | OTHER BACTERIAL |
| A04.3 | 008.04 | Enterohaemorrhagic Escherichia coli infection | OTHER BACTERIAL |
| A04.4 | 008.09 | Other intestinal Escherichia coli infections | OTHER BACTERIAL |
| A04.5 | 008.43 | Campylobacter enteritis | OTHER BACTERIAL |
| A04.6 | 008.44 | Enteritis due to Yersinia enterocolitica | OTHER BACTERIAL |
| A04.7 | 008.45 | Enterocolitis due to Clostridium Difficile | CDIFF |
| A04.8 | 008.49 | Other specified bacterial intestinal infections | NOS |
| A04.9 | 008.5 | Bacterial intestinal infection, unspecified | NOS |
| A05 | 005 | Other bacterial foodborne intoxications, nec | NOS |
| A05.0 | 005.0 | Foodborne staphylococcal intoxication | OTHER BACTERIAL |
| A05.1 | 005.1 | Botulism | OTHER BACTERIAL |
| A05.2 | 005.2 | Foodborne Clostridium perfringens [Clostridium welchii] intoxication | OTHER BACTERIAL |
| A05.3 | 005.4 | Foodborne Vibrio parahaemolyticus intoxication | OTHER BACTERIAL |
| A05.4 |  | Foodborne Bacillus cereus intoxication | OTHER BACTERIAL |
| A05.5 | 005.81 | Foodborne Vibrio vulnificus intoxication | OTHER BACTERIAL |
| A05.8 | 005.89 | Other specified bacterial foodborne intoxications | NOS |
| A05.9 | 005.9 | Bacterial foodborne intoxication, unspecified | NOS |
| A06 | 006 | Amoebiasis | PARASITIC |
| A06.0 | 006.0 | Acute amoebic dysentery | PARASITIC |
| A06.1 | 006.1 | Chronic intestinal amoebiasis | PARASITIC |
| A06.2 | 006.2 | Amoebic nondysenteric colitis | PARASITIC |
| A06.3 |  | Amoeboma of intestine | PARASITIC |
| A06.8 | 006.8 | Amoebic infection of other sites | PARASITIC |
| A06.9 | 006.9 | Amoebiasis, unspecified | PARASITIC |
| A07 | 007 | Other protozoal intestinal diseases (A07.0; A07.1; A07.2; A07.3; A07.4; A07.8; A07.9) | PARASITIC |
| A08 |  | Viral and other specified intestinal infections | NOS |
| A08.0 | 008.61 | Rotaviral enteritis | ROTA |
| A08.1 |  | AGE due to norovirus and other small round viruses | NORO |
| A08.11 | 008.63 | AGE due to norovirus | NORO |
| A08.19 | 008.64 | AGE due to other small round viruses | NORO |
| A08.2 | 008.62 | Adenoviral enteritis | OTHER VIRAL |
| A08.3 |  | Other viral enteritis | NOS |
| A08.31 | 008.65 | Calicivirus enteritis | NORO |
| A08.32 | 008.66 | Astrovirus enteritis | OTHER VIRAL |
| A08.39 | 008.69 | Other viral enteritis | NOS |
| A08.4 |  | Viral intestinal infection, unspecified | NOS |
| A08.5 | 008.8 | Other specified intestinal infections (from A08.5 in ICD-10 2010 to A08.8 in ICD-10 2016) | NOS |
| A08.8 | 008.8 | Other specified intestinal infections (from A08.5 in ICD-10 2010 to A08.8 in ICD-10 2016) | NOS |
| A09 | 009 | Infectious gastroenteritis and colitis, unspecified origin (A09.0; A09.9) | NOS |
| K52.8 | 558.41; 558.42; 558.9 | Other specified noninfective gastroenteritis and colitis | NOS |
| K52.9 | 558.41; 558.42; 558.9 | Unspecified non-infective gastroenteritis and colitis | NOS |
| R19.7 | 787.91 | Diarrhea, unspecified | NOS |
|  | 005.3 | Food poisoning due to other Clostridia | OTHER BACTERIAL |
|  | 005.8 | Other bacterial food poisoning | NOS |
|  | 008 | Intestinal infections due to other organisms | NOS |
|  | 008.0 | Intestinal infection due to Escherichia coli [E. coli] | OTHER BACTERIAL |
|  | 008.00 | Intestinal infection due to E. coli, unspecified | OTHER BACTERIAL |
|  | 008.1 | Arizona group of paracolon bacili | OTHER BACTERIAL |
|  | 008.2 | Aerobacter aerogenes | OTHER BACTERIAL |
|  | 008.3 | Proteus (mirabilis) | OTHER BACTERIAL |
|  | 008.4 | Intestinal Infection due to other specified bacteria | NOS |
|  | 008.41 | Staphylococcus | OTHER BACTERIAL |
|  | 008.42 | Pseudomonas | OTHER BACTERIAL |
|  | 008.46 | Other anaerobes | NOS |
|  | 008.47 | Other gram-negative bacteria | NOS |
|  | 008.6 | Enteritis due to specified virus | NOS |
|  | 008.67 | Enteritis due to enterovirus nec | OTHER VIRAL |

# Supplementary Table 2: Registers that provided national hospital discharge statistics to the present study

| **Country** | **Data Provider** | **Period** | **ICD version** | **Type of data** | **Population coverage** |
| --- | --- | --- | --- | --- | --- |
| **Austria** | National Institute of Statistics | 2004-2014 | ICD10 | Individual | 100% |
| **Cyprus** | Statistical Service of Cyprus (CYSTAT) | 2004-2014 | ICD10 | Individual | 54-58% |
| **Denmark** | Statistics Denmark (Fuldmægtig Danmarks Statistik (DST) Consulting) | 2004-2015 | ICD10 | Aggregated by code group | 100% |
| **England** | NHS Digital | 2004-2015 | ICD10 | Aggregated by code group | 100% |
| **Finland ^a^** | National Institute for Health and Welfare (THL) | 2004-2015 | ICD10 | Aggregated by code group | 100% |
| **Germany** | Federal Statistics Office (DESTATIS) | 2004-2015 | ICD10 | Aggregated by code group | 100% |
| **Hungary** | National Healthcare Centre (AEEK) | 2004-2015 | ICD10 | Aggregated by individual code | 97-99% |
| **Italy** | National Center for Epidemiology, Surveillance and Health Promotion (CNESPS) | 2004-2013 | ICD9 | Individual | 97-99% |
| **Lithuania** | Health Statistics Department, Health information Centre, Institute of Hygiene | 2004-2015 | ICD10 | Individual | 99% |
| **Malta** | Directorate for Health Information and Research, Ministry of Health | 2010-2015 | ICD10 | Individual | 100% |
| **Norway** ^b^ | Norwegian Patient Registry, Directory of Health | 2004-2015 | ICD10 | Aggregated by code group | 100% |
| **Poland** | Center for Monitoring and Analysis of Population Health, National Institute of Public Health | 2004-2015 | ICD10 | Aggregated by individual code | 85-97% |
| **Portugal** | Healthcare Financing Department, Central Administration of the National Health Service (GDH) | 2004-2015 | ICD9 | Individual | 75-76% |
| **Romania** | Centre for Research and Evaluation of Health Services, Public Health School | 2006-2015 | ICD10 | Individual | 100% |
| **Spain** | Institute of Health Information, Ministry of Health, Social Services and Equity | 2004-2014 | ICD9 | Individual | 84-100% |
| **Sweden** | National Board of Health and Welfare, Department of Statistic | 2004-2015 | ICD10 | Individual | 100% |

a) Finland provided data aggregated for the age groups: under 18 years, 18-59 years and 60 years and older. Consequently, data were included in analysis for all ages but excluded from age-specific analysis. b) Data from Norway were excluded from this analysis, since primary (first-listed) and secondary diagnosis were aggregated.

# Supplementary Table 3: Non-included countries in Europe: data provider contacted and reason for exclusion

| **Country** | **Contacted provider** | **Reason** |
| --- | --- | --- |
| Belgium | Health, Official information and services, Belgium Federal Government | Complex authorization process, including a National Privacy Commission |
| Bulgaria | National Center of Public Health and Analyses (**NCPHA**) | No data available/collected at national level |
| Croatia | Croatian Institute of Public Health (HZJZ) | Stalled response from data holder |
| Czech Republic | Institute of Health Information and Statistics (UZIS) | No response from data holder |
| Estonia | National Institute for Health Development (TAI) | No data available/collected at national level |
| France | CNIL (Commission national de l’Informatique et des Libertés) and ATIH (L’Agence technique de l’information sur l’hospitalisation), Ministry of Health | Complex authorization process, including a National Privacy Commission |
| Greece | Not identified |  |
| Iceland | Directorate of Health | Data not accessible to external bodies |
| Ireland | Healthcare Pricing Office (HPO) | Data not accessible to external bodies |
| Latvia | National Health Services (NVD), Ministry of Health | Data not accessible to external bodies |
| Liechtenstein | Not identified |  |
| Luxembourg | Healthnet, National Agency for Health Information (eSanté) | No response from data holder |
| Netherlands | Statistics Netherlands (CBS) | Available only to local research institutions or institutions affiliated to local research institutions |
| Slovakia | Health Policy Institute (HPI) | No data available/collected at national level |
| Slovenia | Ministry of Health (NIJZ) | No response from data holder |
| Switzerland | Federal Statistical Office (FSO) | Data not accessible to external bodies |

# Supplementary Table 4: Mean annual number and mean annual rate of hospital discharges for all-cause acute gastroenteritis (AGE) as first-listed diagnosis in participating European countries between 2004 and 2015

| **Country** | **Annual mean number of discharges for AGE** | **Annual mean discharge rate for AGE**  (/10,000 person-years) |
| --- | --- | --- |
| Austria | 35,557 | 42.7 |
| Cyprus | 982 | 21.3 |
| Denmark | 11,971 | 21.7 |
| England | 82,618 | 15.8 |
| Finland | 12,995 | 24.3 |
| Germany | 321,564 | 39.4 |
| Hungary | 7,035 | 7.2 |
| Italy | 49,972 | 8.6 |
| Lithuania | 17,347 | 55.4 |
| Malta | 1,243 | 29.6 |
| Poland | 105,006 | 29.9 |
| Portugal | 8,614 | 10,9 |
| Romania | 58,656 | 28.8 |
| Spain | 43,856 | 10.8 |
| Sweden | 14,419 | 15.5 |
| **All** | **771,834** | **23.3** |

# Supplementary Table 5: Goodness of fit

| **Country,**  **Age group**  **(years)** | | **Parameter estimates**  **Estimate (standard error)** | | | | | | |  | **Goodness of fit**  **(p-value)** |
| --- | --- | --- | --- | --- | --- | --- | --- | --- | --- | --- |
|  |  | 1000 x α  pt | β1  CDIFF | β2  OTHER BACTERIAL | 0.1 x β3  OTHER  VIRAL | 0.1 x β4  PARASITIC | 100 x β5  ROTA_  < 5 | 10^9 x γ  t x pt |  |  |
| **Austria** | | | | | | | | | | |
|  | < 5 | 9.704 (0.857) | 22.244 (10.139) | - | 0.241 (0.088) | - | 96.793 (9.814) | -482.678 (229.259) |  | 0.354 |
|  | 5-9 | 4.044 (0.210) | - | - | - | - | 7.287 (2.113) | 407.086 (83.990) |  | 0.404 |
|  | 10-17 | 3.228 (0.093) | - | - | - | - | - | 271.994 (43.722) |  | 0.446 |
|  | 18-59 | 2.221 (0.048) | - | - | - | - | - | -118.333 (19.978) |  | 0.430 |
|  | 60-69 | 2.906 (0.093) | - | - | - | - | 2.346 (0.897) | -94.989 (34.632) |  | 0.430 |
|  | 70-79 | 5.666 (0.216) | 1.257 (0.573) | - | - | - | 6.277 (1.923) | -411.788 (87.080) |  | 0.389 |
|  | ≥ 80 | 11.195 (0.458) | 1.478 (0.550) | - | - | - | 25.445 (4.764) | -863.724 (248.463) |  | 0.389 |
| **Denmark** | | | | | | | | | | |
|  | < 5 | 7.527 (0.458) | - | - | - | - | 459.900 (57.204) | -580.369 (187.169) |  | 0.386 |
|  | 5-9 | 1.613 (0.074) | - | - | - | - | 14.373 (5.415) | -111.889 (28.964) |  | 0.427 |
|  | 10-17 | 0.938 (0.043) | - | 1.308 (0.388) | - | - | - | -49.496 (14.033) |  | 0.400 |
|  | 18-59 | 0.837 (0.036) | - | 2.034 (0.439) | - | - | - | 52.969 (9.979) |  | 0.403 |
|  | 60-69 | 1.314 (0.040) | - | - | - | - | 12.177 (2.925) | 55.462 (16.583) |  | 0.473 |
|  | 70-79 | 2.976 (0.084) | 0.382 (0.189) | - | - | - | 18.861 (5.952) | -133.390 (55.686) |  | 0.410 |
|  | ≥ 80 | 6.080 (0.208) | 0.403 (0.205) | - | - | - | 58.590 (16.224) | -419.559 (162.432) |  | 0.388 |
| **England** | | | | | | | | | | |
|  | < 5 | 2.923 (0.190) | - | - | - | - | 474.100 (19.894) | -57.534 (51.899) |  | 0.366 |
|  | 5-9 | 0.677 (0.022) | - | - | - | - | 30.745 (2.143) | 26.575 (8.236) |  | 0.423 |
|  | 10-17 | 0.483 (0.011) | - | - | - | - | 3.199 (0.854) | 11.683 (4.207) |  | 0.423 |
|  | 18-59 | 0.502 (0.017) | - | 1.254 (0.273) | - | - | 2.712 (0.683) | 23.372 (3.179) |  | 0.359 |
|  | 60-69 | 0.979 (0.036) | 0.479 (0.197) | - | - | - | 7.946 (1.313) | 66.506 (6.846) |  | 0.394 |
|  | 70-79 | 1.987 (0.083) | 0.311 (0.136) | - | - | - | 25.280 (3.215) | 113.401 (17.180) |  | 0.393 |
|  | ≥ 80 | 4.723 (0.102) | - | - | - | - | 79.543 (8.574) | 174.847 (38.733) |  | 0.410 |
| **Finland** | | | | | | | | | | |
|  | < 18 | 0.777 (0.108) | - | - | - | - | 362.545 (22.470) | 28.186 (35.553) |  | 0.405 |
|  | 19-59 | 0.833 (0.035) | - | - | - | - | 10.335 (2.725) | -73.704 (11.263) |  | 0.426 |
|  | ≥ 60 | 3.815 (0.332) | - | - | - | - | 294.606 (38.276) | -210.699 (106.417) |  | 0.379 |
| **Germany** | | | | | | | | | | |
|  | < 5 | 8.534 (0.459) | 14.183 (7.146) | - | 0.276 (0.058) | - | 37.026 (3.253) | -1263.783 (172.183) |  | 0.353 |
|  | 5-9 | 3.356 (0.131) | - | - | 0.474 (0.183) | - | 5.405 (0.789) | -173.336 (37.245) |  | 0.382 |
|  | 10-17 | 3.073 (0.089) | 10.453 (5.022) | - | - | - | 1.377 (0.565) | -25.986 (35.310) |  | 0.384 |
|  | 18-59 | 1.244 (0.060) | 4.427 (1.373) | - | - | - | - | 15.145 (24.543) |  | 0.407 |
|  | 60-69 | 1.355 (0.064) | 1.412 (0.439) | - | 0.944 (0.477) | - | 1.163 (0.338) | -11.232 (29.316) |  | 0.365 |
|  | 70-79 | 2.793 (0.149) | 0.882 (0.335) | - | 0.998 (0.447) | - | 3.029 (0.696) | -82.844 (61.171) |  | 0.357 |
|  | ≥ 80 | 5.805 (0.296) | 0.802 (0.307) | - | 2.949 (0.685) | - | 9.811 (2.031) | -319.159 (215.732) |  | 0.359 |
| **Hungary** | | | | | | | | | | |
|  | < 5 | 6.692 (0.293) | - | - | - | - | 261.544 (26.461) | -1220.724 (84.582) |  | 0.394 |
|  | 5-9 | 2.288 (0.112) | - | - | - | - | 42.233 (9.377) | -366.437 (32.507) |  | 0.358 |
|  | 10-17 | 0.905 (0.033) | - | - | - | - | - | -79.858 (12.471) |  | 0.332 |
|  | 18-59 | 0.204 (0.011) | 1.670 (0.417) | 0.991 (0.166) | - | - | - | -37.047 (4.400) |  | 0.445 |
|  | 60-69 | 0.260 (0.014) | - | 0.451 (0.162) | - | - | - | -20.490 (4.124) |  | 0.495 |
|  | 70-79 | 0.466 (0.024) | 0.361 (0.123) | - | - | - | - | -63.267 (14.467) |  | 0.431 |
|  | ≥ 80 | 0.633 (0.048) | 0.278 (0.109) | 0.914 (0.262) | - | - | - | -82.755 (25.404) |  | 0.383 |
| **Italy** | | | | | | | | | | |
|  | < 5 | 5.959 (0.167) | - | - | - | - | 65.874 (5.172) | -1154.246 (57.419) |  | 0.392 |
|  | 5-9 | 1.054 (0.114) | - | 1.053 (0.286) | - | - | 12.441 (1.669) | -193.907 (23.396) |  | 0.370 |
|  | 10-17 | 0.476 (0.043) | - | 1.293 (0.280) | 0.355 (0.170) | - | 2.526 (0.703) | -78.876 (9.147) |  | 0.361 |
|  | 18-59 | 0.240 (0.017) | - | 2.601 (0.334) | - | - | 0.612 (0.242) | -33.327 (3.781) |  | 0.374 |
|  | 60-69 | 0.354 (0.035) | - | 1.929 (0.417) | - | - | 1.852 (0.505) | -42.550 (6.872) |  | 0.367 |
|  | 70-79 | 0.658 (0.064) | 1.163 (0.525) | 1.674 (0.504) | - | - | 2.092 (1.049) | -91.812 (21.930) |  | 0.347 |
|  | ≥ 80 | 0.966 (0.119) | - | 2.554 (0.687) | - | - | 6.547 (1.860) | -19.803 (21.500) |  | 0.330 |
| **Lithuania** | | | | | | | | | | |
|  | < 5 | 25.613 (1.111) | - | - | - | - | 14.331 (3.379) | 986.878 (421.096) |  | 0.386 |
|  | 5-9 | 4.914 (0.312) | - | - | - | - | - | 2154.893 (161.297) |  | 0.413 |
|  | 10-17 | 2.280 (0.168) | - | - | - | - | - | 952.640 (86.789) |  | 0.427 |
|  | 18-59 | 1.173 (0.047) | - | - | - | - | - | -7.404 (19.069) |  | 0.433 |
|  | 60-69 | 1.523 (0.065) | - | - | - | - | - | -54.513 (25.556) |  | 0.466 |
|  | 70-79 | 2.241 (0.095) | - | - | - | - | - | -17.077 (38.246) |  | 0.446 |
|  | ≥ 80 | 2.662 (0.164) | - | - | - | - | 0.906 (0.458) | 68.714 (59.689) |  | 0.384 |
| **Poland** | | | | | | | | | | |
|  | < 5 | 18.162 (0.494) | - | - | - | - | 55.992 (4.599) | -1093.876 (197.679) |  | 0.400 |
|  | 5-9 | 3.318 (0.490) | - | 1.039 (0.381) | - | - | 20.027 (1.855) | 266.906 (85.200) |  | 0.343 |
|  | 10-17 | 0.736 (0.220) | - | 2.802 (0.554) | - | - | 2.593 (0.866) | 437.019 (36.581) |  | 0.356 |
|  | 18-59 | 0.384 (0.026) | - | 1.085 (0.145) | - | - | 0.722 (0.096) | 9.740 (5.381) |  | 0.366 |
|  | 60-69 | 0.886 (0.040) | - | 0.762 (0.219) | - | - | 2.047 (0.212) | -35.332 (9.595) |  | 0.368 |
|  | 70-79 | 1.306 (0.074) | - | 1.382 (0.292) | - | - | 4.441 (0.411) | -6.529 (18.215) |  | 0.391 |
|  | ≥ 80 | 1.955 (0.115) | - | 1.652 (0.401) | - | - | 7.197 (0.628) | 28.813 (28.533) |  | 0.343 |
| **Portugal** | | | | | | | | | | |
|  | < 5 | 7.775 (0.338) | - | - | 0.416 (0.162) | - | 133.266 (11.545) | -1508.562 (108.784) |  | 0.366 |
|  | 5-9 | 2.094 (0.151) | - | 1.639 (0.282) | 0.486 (0.238) | - | 12.347 (2.959) | -302.936 (40.056) |  | 0.427 |
|  | 10-17 | 0.679 (0.047) | - | 1.614 (0.251) | - | - | - | -0.283 (16.019) |  | 0.412 |
|  | 18-59 | 0.136 (0.009) | - | 3.330 (0.308) | - | - | - | 3.063 (2.786) |  | 0.372 |
|  | 60-69 | 0.439 (0.025) | - | 1.415 (0.389) | - | - | - | 5.136 (8.595) |  | 0.426 |
|  | 70-79 | 0.812 (0.045) | - | 2.971 (0.521) | - | - | - | 43.448 (14.919) |  | 0.380 |
|  | ≥ 80 | 2.418 (0.079) | - | - | - | - | - | 94.130 (32.111) |  | 0.357 |
| **Romania** | | | | | | | | | | |
|  | < 5 | 7.314 (2.127) | - | 13.195 (1.477) | - | - | - | 1945.733 (531.535) |  | 0.387 |
|  | 5-9 | 0.507 (0.328) | - | 10.548 (1.252) | - | - | - | 883.539 (115.652) |  | 0.383 |
|  | 10-17 | -0.268 (0.131) | - | 9.324 (0.898) | - | - | - | 614.708 (45.508) |  | 0.392 |
|  | 18-59 | 0.504 (0.079) | - | 4.939 (0.588) | - | - | - | -11.010 (20.860) |  | 0.384 |
|  | 60-69 | 0.847 (0.124) | - | 4.651 (0.849) | - | - | - | 5.598 (33.279) |  | 0.379 |
|  | 70-79 | 0.783 (0.133) | - | 7.001 (0.867) | - | - | - | 86.637 (40.538) |  | 0.389 |
|  | ≥ 80 | 0.768 (0.155) | - | 5.818 (1.082) | - | - | - | 100.634 (50.578) |  | 0.359 |
| **Spain** | | | | | | | | | | |
|  | < 5 | 3.845 (0.272) | - | - | 0.651 (0.130) | - | 59.253 (5.393) | -821.071 (67.846) |  | 0.343 |
|  | 5-9 | 0.971 (0.043) | - | 0.523 (0.102) | - | - | - | -102.755 (11.783) |  | 0.387 |
|  | 10-17 | 0.379 (0.016) | - | 0.558 (0.102) | - | - | - | -8.868 (5.157) |  | 0.400 |
|  | 18-59 | 0.234 (0.007) | - | 0.872 (0.102) | - | - | - | 10.516 (2.055) |  | 0.388 |
|  | 60-69 | 0.660 (0.032) | - | 0.472 (0.175) | - | - | 1.299 (0.523) | 17.884 (6.836) |  | 0.372 |
|  | 70-79 | 1.437 (0.032) | - | - | - | - | - | 66.619 (14.510) |  | 0.434 |
|  | ≥ 80 | 2.685 (0.073) | - | - | 2.101 (1.069) | - | 6.715 (1.859) | 124.307 (29.090) |  | 0.373 |
| **Sweden** | | | | | | | | | | |
|  | < 5 | 2.310 (0.224) | - | - | 0.674 (0.181) | - | 490.768 (29.364) | -403.338 (72.414) |  | 0.352 |
|  | 5-9 | 0.511 (0.037) | - | - | - | - | 62.484 (3.654) | -48.179 (13.216) |  | 0.327 |
|  | 10-17 | 0.300 (0.016) | - | - | - | - | 4.609 (1.001) | -13.594 (5.918) |  | 0.282 |
|  | 18-59 | 0.459 (0.020) | - | 0.319 (0.117) | - | - | 3.634 (0.752) | -15.881 (4.216) |  | 0.353 |
|  | 60-69 | 0.824 (0.027) | - | - | - | - | 10.478 (1.604) | -22.968 (9.604) |  | 0.378 |
|  | 70-79 | 1.384 (0.098) | 0.375 (0.152) | - | 0.685 (0.346) | - | 27.976 (3.122) | -91.187 (17.689) |  | 0.341 |
|  | ≥ 80 | 3.280 (0.148) | - | - | 2.299 (0.723) | - | 112.438 (11.029) | -165.860 (53.346) |  | 0.411 |

# Supplementary Table 6: List of Data Providers / Requirements

| Country | Name | Contact person/ Email | Telephone No. | Regarding dissemination | Requirements from data provider |
| --- | --- | --- | --- | --- | --- |
| Austria | Bundesanstalt Statistik Österreich / National Institute of Statistic, Austria | Jeannette KLIMONT [Jeannette.Klimont@statistik.gv.at](mailto:Jeannette.Klimont@statistik.gv.at)  [office@statistik.gv.at](mailto:office@statistik.gv.at) | +43 (1) 71128-0 | No specific provisions | No specific provisions  Data should not be used for other purposes |
| Cyprus | Statistical Service of Cyprus | Isidoros Kypridakis [ikypridakis@cystat.mof.gov.cy](mailto:ikypridakis@cystat.mof.gov.cy)  [enquiries@cystat.mof.gov.cy](mailto:enquiries@cystat.mof.gov.cy) | +35722602166 | No specific provisions | - Data should not be disclosed to 3rd parties - Destroy data and copies once utilized for the research purpose - Data should not be used for other purposes |
| Denmark | Fuldmægtig  Danmarks Statistik (DST) Consulting | Carsten Barsøe  [CRB@dst.dk](mailto:CRB@dst.dk) | 39 17 35 23 | Statistics Denmark must be listed as data source | - Data should not be disclosed to 3rd parties - Data should not be used for other purposes |
| England | NHS Digital | HES, Hscic (NHS DIGITAL) [hes.questions@nhs.net](mailto:hes.questions@nhs.net) | +44300 303 5678 | No specific provisions | - Data should not be used for other purposes - Destroy data and copies once utilized for the research purpose |
| Finland | Terveyden Ja Hyvinvoinnin Laitos (THL) / National Institute For Health And Welfare | Rasilainen Jouni [jouni.rasilainen@thl.fi](mailto:jouni.rasilainen@thl.fi)  [sotkanet@thl.fi](mailto:sotkanet@thl.fi)  [jutta.jarvelin@thl.fi](mailto:jutta.jarvelin@thl.fi) | +358 29 524 7204 | No specific provisions | Data should not be used for other purposes |
| Germany | Statistisches Bundesamt (DESTATIS)  Zentraler Auskunftsdienst | Ralf Müller  [info@destatis.de](mailto:info@destatis.de) | +49 (0) 611 75 2405 | No specific provisions | Data should not be used for other purposes |
| Hungary | Állami Egészségügyi Ellátó Központ / National Healthcare Center | Surján György [surjan.gyorgy@aeek.hu](mailto:surjan.gyorgy@aeek.hu)  [aeek@aeek.hu](mailto:aeek@aeek.hu) | +361 356 1522 | No specific provisions | - Data should not be disclosed to 3rd parties - Data should not be used for other purposes |
| Italy | Centro Nazionale di Epidemiologia, Sorveglianza e Promozione della Salute (CNESPS) - Istituto Superiore di Sanità / Health Institute | Rizzo Caterina  [caterina.rizzo@iss.it](mailto:caterina.rizzo@iss.it) | +39 06 4990 1 | No specific provisions | Data should not be used for other purposes |
| Lithuania | Health Statistics Department  Health information Centre,  Institute of Hygiene, Lithuania | Rita Gaidelyte  [rita.gaidelyte@hi.lt](mailto:rita.gaidelyte@hi.lt)  [institutas@hi.lt](mailto:institutas@hi.lt) | +370 52773303 | No specific provisions | Data should not be used for other purposes |
| Malta | Directorate for Health Information and Research – Ministry of Health | Distefano Alexandra [alexandra.distefano@gov.mt](mailto:alexandra.distefano@gov.mt)  Calleja Neville [neville.calleja@gov.mt](mailto:neville.calleja@gov.mt) | +356 25599262 | DHI will be acknowledged in any publications / presentations based on the data provided  *“We would like to have access to the results especially if they will be presented at a country level where Malta will be identified”* | - Data should not be disclosed to 3rd parties - Destroy data and copies once utilized for the research purpose - Data should not be used for other purposes |
| Norway | Norwegian Patient Registry (NPR)  Norwegian Directory of Health | Hilde Kjeldstad Berg [Hilde.Kjeldstad.Berg@helsedir.no](mailto:Hilde.Kjeldstad.Berg@helsedir.no)  [NPR.felles@helsedir.no](mailto:NPR.felles@helsedir.no) | +47 47 25 72 22 | - NPR must be listed as the source - The following disclaimer should be included in any publication:   Disclaimer  “Data from the Norwegian Patient Registry has been used in this publication. The interpretation and reporting of these data are the sole responsibility of the authors, and no endorsement by the Norwegian Patient Registry is intended nor should be inferred” | - Data should not be disclosed to 3rd parties - Data should not be used for other purposes |
| Poland | Zakład Centrum Monitorowania i Analiz Stanu Zdrowia Ludności Narodowy Instytut Zdrowia Publicznego / Center for Monitoring and Analysis of Population Health- National Institute of Public Health | Goryński Paweł  [pawel@pzh.gov.pl](mailto:pawel@pzh.gov.pl)  [pzh@pzh.gov.pl](mailto:pzh@pzh.gov.pl) | 22 54 21 236 | No specific provisions | - Data should not be disclosed to 3rd parties - Data should not be used for other purposes |
| Portugal | Base de Dados Nacional de Grupo de Diagnóstico Homogéneo (GDH)  Healthcare Financing Department. Central administration of the National Health Service. Portugal | Cláudia Borges  [cborges@acss.min-saude.pt](mailto:cborges@acss.min-saude.pt)  [geral@acss.min-saude.pt](mailto:geral@acss.min-saude.pt) | +351 21 792 55 22 | - A copy of any publications/presentations will be submitted to GDH - GDH must be listed as source | - Data should not be disclosed to 3rd parties - Data should not be used for other purposes |
| Romania | Centre for Research  and Evaluation of Health Services. Public Health School, Bucarest | Mihnea Dosius [MDosius@snspms.ro](mailto:MDosius@snspms.ro) | +021 252 04 25 | No specific provisions | Data should not be used for other purposes |
| Spain | Instituto de Información Sanitaria/ Institute of Health Information  Ministerio Sanidad, Servicios Sociales e Igualdad / Ministry of Health, Social Services and Equity | [icmbd@msssi.es](mailto:icmbd@msssi.es) | +34 91 596 15 65 | - Copy of publication/s should be sent to data provider - Instituto de Informacion Sanitaria cited as data source | - Destroy data and copies once utilized for the research purpose - Data should not be disclosed to 3rd parties |
| Sweden | SOCIALSTYRELSEN /National Board of Health and Welfare Avdelningen för statistik och jämförelser/ Department of Statistics | Tomas Wänskä [tomas.wanska@socialstyrelsen.se](mailto:tomas.wanska@socialstyrelsen.se) statistikdatabas statistikdatabas@socialstyrelsen.se | 075-247 30 00 | No specific provisions | Data should not be used for other purposes |
